# Generalized Open Quantum Walks on Apollonian Networks

**DOI:** 10.1371/journal.pone.0130967

**Published:** 2015-07-15

**Authors:** Łukasz Pawela, Piotr Gawron, Jarosław Adam Miszczak, Przemysław Sadowski

**Affiliations:** Institute of Theoretical and Applied Informatics, Polish Academy of Sciences, Bałtycka 5, 44-100 Gliwice, Poland; University of Namur, BELGIUM

## Abstract

We introduce the model of generalized open quantum walks on networks using the Transition Operation Matrices formalism. We focus our analysis on the mean first passage time and the average return time in Apollonian networks. These results differ significantly from a classical walk on these networks. We show a comparison of the classical and quantum behaviour of walks on these networks.

## Introduction

Understanding the information flow in classical and quantum networks is crucial for the comprehension of many phenomena in physics, social sciences and biology [1–3]. Real-world networks are usually small-world and scale-free. An important example of networks which posses both of these properties are Apollonian networks.

Random walks provide a useful model for studying the behaviour of agents in complex networks [4–10]. In particular in [11] it was shown that for the class of finite connected undirected networks, walks for which probability of leaving a node is reciprocal of its degree, have a fixed average return time (ART). Mean first passage time (MFPT) and ART in the case of deterministic and random Apollonian networks have been studied by Huang *et al.* [12].

In this paper we investigate the behaviour of quantum walks of the class of Apollonian networks. Using the concept of generalised open quantum walks (GOQW) we introduce the definition of MFPT in the quantum case. The notion of GOQW allows us the consideration of a broader class of walks compared to the open quantum walks introduced in [13–20]. The main limitation in using the open quantum walks is the lack of flexibility in assigning the weights to the edges.

The motivation for performing the research presented in this paper was to study coin-less quantum walks on undirected graphs, with weights on edges. We also assume that for each edge its weight in one direction is not necessary related with the weight in the other direction. In the usual setting [21] quantum walks are defined by a Hamiltonian derived from the adjacency matrix. Due to the hermiticity of the Hamiltonian the intensity of transition from vertex *i* to vertex *j* is related to the intensity of transition from *j* to *i*. One way to overcome this limitation is to use the technique introduced by Szegedy [22]. In this paper we propose an alternative approach by introducing generalized open quantum walks. Moreover, we apply this formalism to extend the analysis performed in [11], where the authors studied the relation between the degree of the vertex and mean first passage time of a Markov process.

The paper is organized as follows. In the next section we introduce basic concepts concerning the presented work such as the notions of quantum mechanics, a generalization of the open quantum walk (OQW) model and the notion of quantum transition operation matrix (TOM). Subsequently we provide the methods of constructing the generalized open quantum walks on Apollonian networks and discuss some particular cases. Finally, we provide the concluding remarks and suggest a direction for further work.

## Preliminaries

### Apollonian networks

Apollonian networks are named after Apollonius of Perga, who introduced the problem of space filling by packing spheres [23, 24]. The concept of Apollonian networks was introduced by Andrade *et al.* [25] and by Doye and Massen in [26]. In [25] it was shown that it can be used to describe force chains in polydisperse granular packings, whilst in [26] topological and spatial properties of such networks are characterized and their application as model for networks of connected energy minima is discussed.

**Fig 1:**
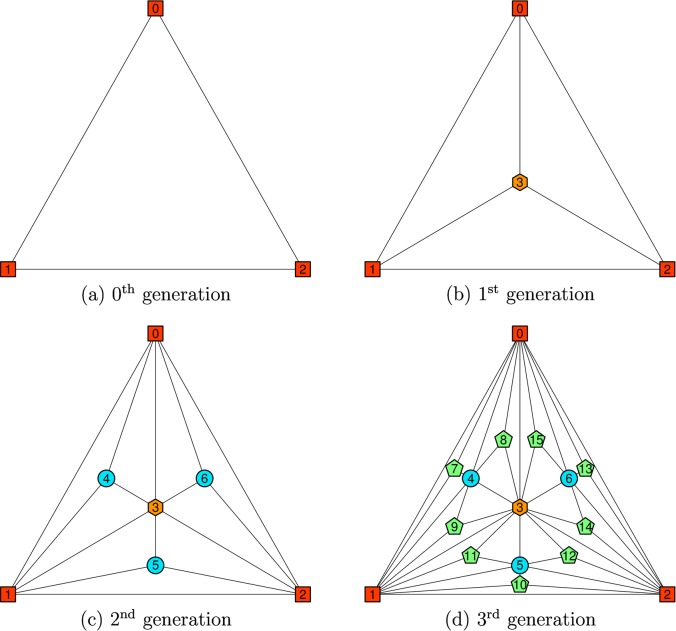
An illustration of the construction of an Apollonian network. Red squares illustrate the nodes in the 0^th^ generation, an orange hexagon in the 1^st^ generation, blue circles in the 2^nd^ generation and green pentagons in the 3^rd^ generation.

The construction of a regular Apollonian network can be done by a recursive procedure. At first, a complete 3-vertex graph is created, we call it the 0^th^ generation Apollonian network. In order to obtain the next generation network new nodes are inserted in the middle of each of the triangles in the graph. Each of the new vertices is associated with three new edges connecting it to the vertices of the corresponding triangle. Apollonian networks of generations zero to three resulting from the algorithm are presented in the Fig 1.

Apollonian networks display some properties which make them a very useful tool for studying effects in large complex networks. In particular they have the property of being scale-free and small-world. They can be also embedded in Euclidean lattice, and show space filling and matching graph properties.

Apollonian networks have been used in various areas of science. In particular, Andrade and Herrmann [27] and Serva *et al.* [28] investigated the properties of Ising models on Apollonian network. It was also suggested that Apollonian networks can be harnessed to mimic a behaviour of neuronal systems in the brain [29]. Random Apollonian networks [30] were introduced as a model for real-world planar graphs. Their high-dimensional generalizations were also proposed in [31]. The properties of random Apollonian networks were studied in [32] in the context of web graphs.

Various researchers considered walks on Apollonian networks. For example Huang *et al.* [12] studied classical random walks on these networks. Random walks on Apollonian networks with defects were considered by Zang *et al.* [33].

Discrete time quantum walks on Apollonian networks were studied by Souza and Andrade in [34], where a comparison of the introduced model with its classical counterpart was provided. Xu *et al.* [35] studied the properties of coherent exciton transport on Apollonian networks with dynamics modelled by continuous-time quantum walks. Finally, Sadowski [36] has recently provided an efficient implementation of the quantum search algorithm exploiting the structure of Apollonian networks.

In all of these papers Apollonian networks served as a useful tool for studying properties of various walk models. We utilise Apollonian networks for the same purpose. However, the models considered in the above-mentioned papers are not directly connected with the OQW model and thus the results are not directly comparable. In particular, the aforementioned papers focus on unitary system dynamics, whereas our model allows for implementation of general quantum operations.

### Open quantum walks

Following [15] we recall the notion of Open Quantum Walks. The model of the open quantum walk was introduced by Attal *et al.* [13] (see also [37]). In order to describe the model, we consider a walk on a graph with the set of vertices *V* and directed edges {(*i*, *j*): *i*, *j* ∈ *V*}. The dynamics on the graph is described by the space of states 𝓗_2_ = ℂ^*V*^ with the orthonormal basis ${\left\{|i\rangle \right\}}_{i=0}^{|V|-1}$. We describe an internal degree of freedom of the walker by attaching a Hilbert space 𝓗_1_ to each vertex of the graph. Hence, the state of the quantum walker is described by the element of the space 𝓛(𝓗_1_ ⊗ 𝓗_2_).

Let us imagine a single quantum particle wandering through the vertices of a graph. In discrete moments of time, the particle hops from one vertex *i* to another vertex *j*. With each transition the quantum state of the particle is changed by a quantum operation associated with the edge (*i*, *j*). With each step the particle can, but does not have to, hop to all neighbours of vertex *i*. Thus, after several steps the particle may become “smeared” over the vertices of the graph.

### Quantum states and quantum channels

In the following we recall the standard notions of quantum mechanics that are essential for understanding the content of this paper.

**Definition 1** *Linear Hermitian operator *ρ* ∈ 𝓛(𝓗) that is positive semi-definite (*ρ* ≥ 0) and has a trace lesser or equal to one (Tr(*ρ*) ≤ 1) is called a sub-normalized quantum state. A set of sub-normalized quantum states acting on 𝓗 will be denoted as Ω_≤_(𝓗)*.

**Definition 2** *If a sub-normalized quantum state has a unit trace (Tr*ρ* = 1), then it is called a quantum state. A set of quantum states acting on 𝓗 will be denoted as Ω(𝓗)*.

**Definition 3** *A linear map Φ:𝓛(𝓗_*I*_) → 𝓛(𝓗_*O*_) is completely positive (CP) iff for some *K* it can be written as*

Φ(ρ)=∑k=1KEk†ρEk†, (1)

*where *E*_*k*_ ∈ 𝓛(𝓗_*I*_, 𝓗_*O*_) are called Kraus operators and *ρ* ∈ 𝓛(𝓗_*I*_)*.

**Definition 4** *A linear map Φ:𝓛(𝓗_*I*_) → 𝓛(𝓗_*O*_) is trace non-increasing (TNI) iff*

$$\begin{array}{c} \text{Tr}\left(\Phi \left(\rho \right)\right)\leq 1,\forall \rho \in \Omega \left({𝓗}_{I}\right). \end{array}$$ (2)

**Definition 5** *A linear map Φ:𝓛(𝓗_*I*_) → 𝓛(𝓗_*O*_) is trace preserving (TP) iff*

$$\begin{array}{c} \text{Tr}\left(\Phi \left(\rho \right)\right)=1,\forall \rho \in \Omega \left({𝓗}_{I}\right). \end{array}$$ (3)

**Definition 6** *A linear map Φ that is completely positive and trace non-increasing (CP-TNI) is called a quantum operation*.

**Definition 7** *A linear map Φ that is completely positive and trace preserving (CP-TP) is called a quantum channel*.

**Remark 1** *CP-TNI map given by Kraus operators* *E*_*k*_ ∈ 𝓛(𝓗_*I*_, 𝓗_*O*_) *fulfills the condition* $\sum_{k}E_{k}^{†}E_{k}\leq {𝟙}_{{𝓗}_{O}}$, *accordingly CP-TP fulfills the condition* $\sum_{k}E_{k}^{†}E_{k}={𝟙}_{{𝓗}_{O}}$ [38].

**Definition 8** *Mapping* *μ*:*O* → *F* *from a finite set of measurement outcomes* $O={\left\{o_{i}\right\}}_{i=1}^{N}$ *into a set of measurement operators* $F={\left\{A_{i}:A_{i}\in 𝓛({𝓗}_{I},{𝓗}_{O})\right\}}_{i=1}^{N}$ *that fulfills the following relation*

$$\begin{array}{c} \sum_{i=1}^{N}A_{i}^{†}A_{i}={𝟙}_{{𝓗}_{O}} \end{array}$$ (4)

*is called a quantum measurement. Probability* *p*_*i*_ *of measuring the outcome* *o*_*i*_ *in the state* *ρ* *is given by* $p_{i}=\text{Tr}(A_{i}\rho A_{i}^{†})$. *Given the measurement outcome* *o*_*i*_ *the sub-normalized quantum state after the measurement* *μ* *is given by* $\rho_{o_{i}}=A_{i}\rho A_{i}^{†}$.

In this work we limit ourselves to square projective orthonormal measurement operators *i.e.* *A*_*i*_ ∈ 𝓛(𝓗_*I*_), $A_{i}^{2}=A_{i}$ for all *i* ∈ 1, …, *N* and for all *i*, *j* ∈ 1…, *N* *A*_*i*_ *A*_*j*_ = *δ*_*ij*_ *A*_*i*_.

### Generalized open quantum walks

To formally describe the dynamics of the generalized open quantum walk we introduce a quantum operation 𝓔_*ij*_, for each edge (*j*, *i*). This operation describes the change in the internal degree of freedom of the walker due to the move from vertex *j* to vertex *i*. We impose the limitation that the sum of all quantum operations associated with the edges leaving vertex *j* form a quantum channel.

To describe generalized open quantum walks we use the notion of Transition Operation Matrices, introduced in [39], which provides a generalization of stochastic matrices.

**Definition 9** *Sub-Transition Operation Matrix (sub-TOM)* $𝓔={\left\{{𝓔}_{ij}\right\}}_{i,j=1}^{M,N}$ *is a matrix of completely-positive trace non-increasing (CP-TNI) maps such that*

$$\begin{array}{c} \forall_{1\leq j\leq N}\sum_{i=1}^{M}{𝓔}_{ij}=\Phi_{j}, \end{array}$$ (5)

*where Φ_*j*_ are completely positive trace non-increasing (CP-TNI) maps*.

**Definition 10** *Transition Operation Matrix (TOM) is a sub-TOM with every Φ_*j*_ being a completely positive trace preserving (CP-TP) map*.

In this work we will only consider square TOMs, therefore in what follows we assume *M* = *N*. For the sake of simplicity we assume that all operators 𝓔_*ij*_:𝓛(𝓗_1_) → 𝓛(𝓗_1_) act on qudits of dimension dim𝓗_1_ and produce qudits of the same dimension.

**Remark 2** *If* $𝓔={\left\{{𝓔}_{ij}\right\}}_{i,j=1}^{M,K}$ *and* $𝓕={\left\{{𝓕}_{ij}\right\}}_{i,j=1}^{K,N}$ *are TOMs, then their product* 𝓖 = 𝓔𝓕 *is also a TOM such that* ${𝓖}_{ij}=\sum_{k=1}^{K}{𝓔}_{ik}{𝓕}_{kj}$ [39]. *Accordingly, a product of two sub-TOMs is also a sub-TOM*.

With TOM 𝓔 one can associate a Quantum Markov chain according to the following definition.

**Definition 11** *Quantum Markov chain is a finite directed graph* *G* = (*E*, *V*) *labelled by* 𝓔_*ij*_ *for* *e* ∈ *E* *and by zero operator for* $e\in \bar{E}$, *with* *e* ∈ *V* × *V*.

Quantum Markov chain can be represented as *N* × *N* TOM, where *N* = ∣*V*∣. The state of quantum Markov chain is given by a vector state defined as follows.

**Definition 12** *Sub-vector state is a column vector* *α* = (*α*_1_, *α*_2_, …, *α*_*N*_)^*T*^ *such that* *α*_*i*_ *are sub-normalized quantum states* *i.e.* *α*_*i*_ ∈ Ω_≤_(𝓗), *and* $\sum_{i=1}^{N}\alpha_{i}\in \Omega_{\leq }(𝓗)$ *is a sub-normalised quantum state*.

**Definition 13** *Vector state is a sub-vector state for which* $\sum_{i=1}^{N}\alpha_{i}\in \Omega (𝓗)$ *is a quantum state*.

**Fig 2:**
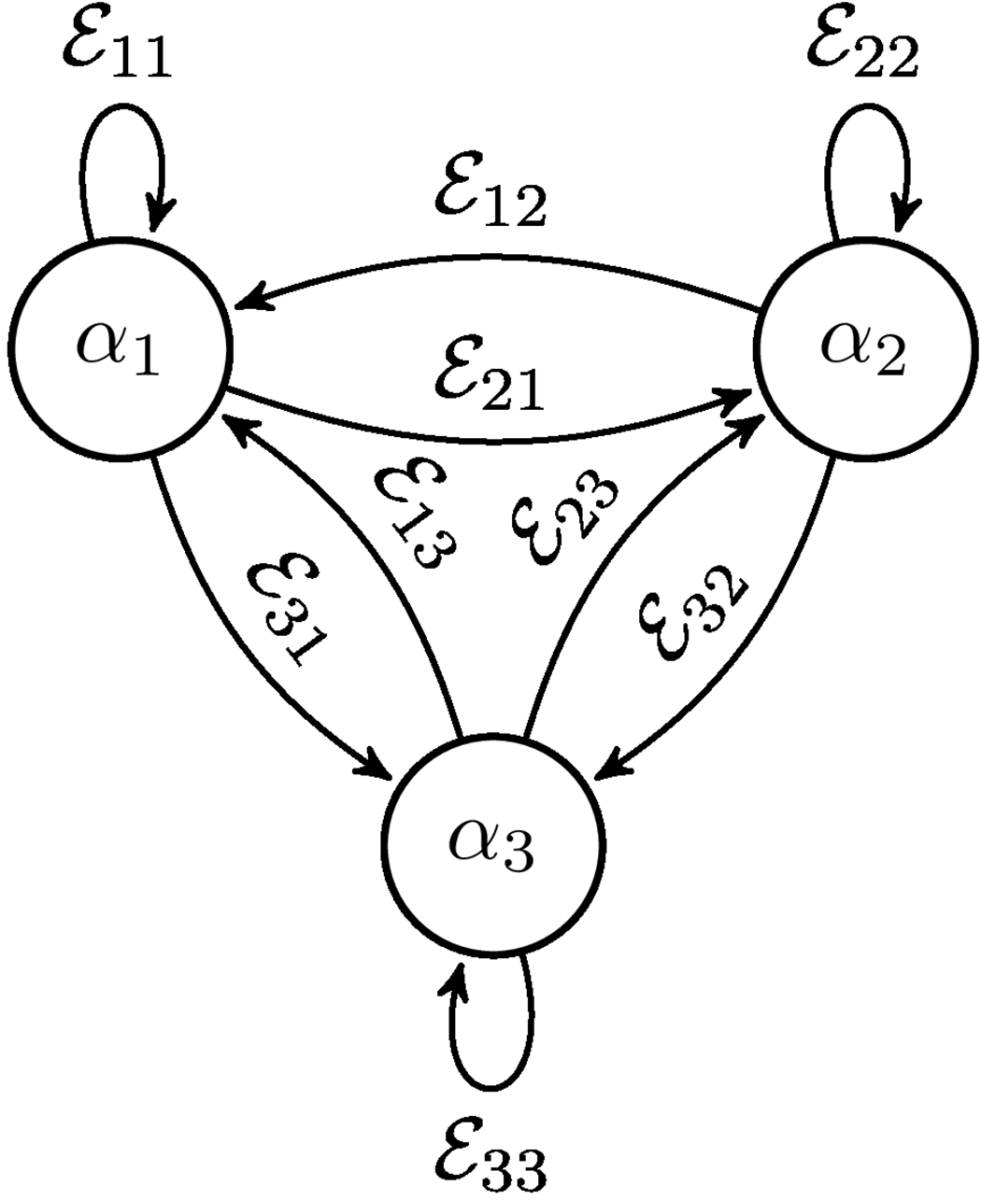
An example of a three state TOM $𝓔=\left[\begin{array}{ccc} {𝓔}_{11} & {𝓔}_{12} & {𝓔}_{13}\\ {𝓔}_{21} & {𝓔}_{22} & {𝓔}_{23}\\ {𝓔}_{31} & {𝓔}_{32} & {𝓔}_{33} \end{array}\right]$. Here *α*_*i*_-s in vertices denote sub-normalized quantum states associated with respective vertices at the given moment of time, therefore the state of the OQW can be described by a vector state *α* = (*α*_1_, *α*_2_, *α*_3_)^*T*^.

Action 𝓔(*α* ^(*t*)^) of (sub-)TOM 𝓔 on a (sub-)vector state *α* ^(*t*)^ at moment *t* produces (sub-)vector state *α* ^(*t*+1)^ at moment *t*+1. This action is obtained in the following way: $\alpha_{i}^{\left(t+1\right)}=\sum_{j=1}^{N}{𝓔}_{ij}(\alpha_{j}^{\left(t\right)})$. An example of a graph associated with a TOM is presented in Fig 2.

One should note that, in the case of one-dimensional internal state space, dim𝓗_1_ = 1, the operators 𝓔_*ij*_ become real numbers and form a stochastic matrix and thus the introduced chain is equivalent to the classical Markov chain.

### TOMs as quantum channels

Let $𝓔={\left\{{𝓔}_{ij}\right\}}_{i=1,j=1}^{N,N}$ be a TOM of dimensions *N* × *N* with elements acting on 𝓛(𝓗_1_). Let each of TOM’s elements 𝓔_*ij*_:𝓛(𝓗_1_) → 𝓛(𝓗_1_) have Kraus operators ${\left\{E_{k_{ij}}\right\}}_{k_{ij}=1}^{K_{ij}}$, where *E*_*k*_*ij*__ ∈ 𝓛(𝓗_1_) and *K*_*ij*_ ∈ ℕ_+_, therefore the action of the elements is given by ${𝓔}_{ij}(\cdot )=\sum_{k_{ij}=1}^{K_{ij}}E_{k_{ij}ij}\cdot E_{k_{ij}ij}^{†}$.

Let us construct the set of operators ${\left\{{\hat{E}}_{k_{ij}ij}\right\}}_{k_{ij}=1,i=1,j=1}^{K_{ij},N,N}$ ${\hat{E}}_{k_{ij}ij}\in 𝓛({𝓗}_{1}⊗{𝓗}_{2})$ in the form ${\hat{E}}_{k_{ij}ij}=E_{k_{ij}ij}⊗|i\rangle \langle j|$, where ${\left\{|i\rangle \right\}}_{i=1}^{N}$ and ${\left\{|j\rangle \right\}}_{j=1}^{N}$ span computational orthonormal bases in 𝓗_1_.

**Definition 14** *A linear map* Φ_𝓔_:𝓛(𝓗_1_ ⊗ 𝓗_2_) → 𝓛(𝓗_1_ ⊗ 𝓗_2_) *associated with TOM 𝓔 is defined by the set of operators* ${\left\{{\hat{E}}_{k_{ij}ij}\right\}}_{k_{ij}=1,i=1,j=1}^{K_{ij},N,N}$.

In what follows we show that, if map Φ_𝓔_ is associated with a TOM 𝓔, then it is CP-TP.

**Proposition 1** *If a set of operators* ${\left\{E_{k_{ij}ij}\right\}}_{k_{ij}=1,i=1,j=1}^{K_{ij},N,N}$ *forms a TOM then the set of operators* ${\left\{{\hat{E}}_{k_{ij}ij}\right\}}_{k_{ij}=1,i=1,j=1}^{K_{ij},N,N}$ *forms a quantum channel*.

**Proof.** To prove this claim it is sufficient to show that operators ${\hat{E}}_{k_{ij}}$ fulfill the completeness relation.

$$\begin{array}{ccc} \sum_{i=1}^{N}\sum_{j=1}^{N}\sum_{k=1}^{K_{ij}}{\hat{E}}_{k_{ij}ij}^{†}{\hat{E}}_{k_{ij}ij} & = & \sum_{i=1}^{N}\sum_{j=1}^{N}\sum_{k=1}^{K_{ij}}{\left(E_{k_{ij}ij}⊗|i\rangle \left\langle j\right|\right)}^{†}\left(E_{k_{ij}ij}⊗|i\rangle \left\langle j\right|\right)\\ & = & \sum_{i=1}^{N}\sum_{j=1}^{N}\sum_{k=1}^{K_{ij}}E_{k_{ij}ij}^{†}E_{k_{ij}ij}⊗\left|j\rangle \langle j\right|\\ & = & \sum_{j=1}^{N}(\sum_{i=1}^{N}\sum_{k=1}^{K_{ij}}E_{k_{ij}ij}^{†}E_{k_{ij}ij})⊗\left|j\rangle \langle j\right|\\ & = & \sum_{j=1}^{N}{𝟙}_{{𝓗}_{1}}⊗\left|j\rangle \langle j\right|\\ & = & {𝟙}_{{𝓗}_{1}⊗{𝓗}_{2}}. \end{array}$$

**Theorem 1** *Let* *α* = (*α*_1_, …, *α*_*j*_, …, *α*_*N*_)^*T*^ *be a vector state. With* *α* *we associate a block diagonal quantum state*

$$\begin{array}{c} \rho_{\alpha }=\sum_{j=1}^{N}\alpha_{j}⊗\left|j\rangle \langle j\right|\in \Omega \left({𝓗}_{1}⊗{𝓗}_{2}\right), \end{array}$$ (6)

*where* *N* = dim𝓗_2_. *Accordingly let* *β* = (*β*_1_, …, *β*_*i*_, …, *β*_*N*_)^*T*^ *be a vector state with an associated state*

$$\begin{array}{c} \rho_{\beta }=\sum_{i=1}^{N}\beta_{i}⊗\left|i\rangle \langle i\right|\in \Omega \left({𝓗}_{1}⊗{𝓗}_{2}\right). \end{array}$$ (7)

*Let* Φ_𝓔_ *be a quantum channel associated with TOM* 𝓔 *and* *β* = 𝓔(*α*), *then*

$$\begin{array}{c} \rho_{\beta }=\Phi_{𝓔}\left(\rho_{\alpha }\right). \end{array}$$ (8)

**Proof.**

$$\begin{array}{ccc} \Phi_{𝓔}\left(\rho_{\alpha }\right) & = & \sum_{i=1}^{N}\sum_{j=1}^{N}\sum_{k_{ij}=1}^{K_{ij}}{\hat{E}}_{k_{ij}ij}\rho_{\alpha }{\hat{E}}_{k_{ij}ij}^{†}\\ & = & \sum_{i=1}^{N}\sum_{j=1}^{N}\sum_{k_{ij}=1}^{K_{ij}}\left(E_{k_{ij}ij}⊗|i\rangle \left\langle j\right|\right)\left(\alpha_{j}⊗|j\right\rangle \left\langle j|\right){\left(E_{k_{ij}ij}⊗|i\rangle \left\langle j\right|\right)}^{†}\\ & = & \sum_{i=1}^{N}\sum_{j=1}^{N}\sum_{k_{ij}=1}^{K_{ij}}E_{k_{ij}ij}\alpha_{j}E_{k_{ij}ij}^{†}⊗\left|i\rangle \langle i\right|\\ & = & \sum_{i=1}^{N}\sum_{j=1}^{N}{𝓔}_{ij}\left(\alpha_{j}\right)⊗\left|i\rangle \langle i\right|=\sum_{i=1}^{N}\beta_{i}⊗\left|i\rangle \langle i\right|\\ & = & \rho_{\beta } \end{array}$$

**Remark 3** *Generalized open quantum walks coincide with open quantum walks introduced in [13] if all the operations have Kraus rank equal to one i.e. can be described with a single Kraus operator*.

### Mean first passage time

There is a number of random walk properties that one can consider in order to analyse walk behaviour. In this paper we focus on the properties commonly examined in the case of classical homogeneous random walks *i.e.* walks with transition probabilities evenly distributed and equal to 1/*d* for each vertex of degree *d*. In particular, we study the mean first passage time and the average return time. The former one describes the average time it takes to make a move between two fixed nodes.

**Definition 15** *The mean first passage time (MFPT) from vertex* *i* *to vertex* *j* *is defined as the average time for the walker to reach vertex* *j* *starting from vertex* *i*:

$$\begin{array}{c} T_{ij}=\sum_{t=1}^{\infty }tP_{ij}\left(t\right), \end{array}$$ (9)

*where* *P*_*ij*_(*t*) *is the first passage probability from* *i* *to* *j* *after time* *t*.

**Definition 16** *The average return time (ART)* *T*_*ii*_ *is the mean first passage time from vertex* *i* *to itself*.

It has been shown that, in the case of homogeneous classical random walks, the ART does not depend on the structure of the network, but only on the degree of the vertex [11]. More precisely, the ART in this case is given by:

$$\begin{array}{c} T_{ii}=\frac{\sum_{j=1}^{N}d_{j}}{d_{i}}, \end{array}$$ (10)

where *d*_*j*_ denotes the degree of the *j* ^th^ vertex. On the other hand, the MFPT depends on the structure of the network and *T*_*ij*_ do not need be equal to *T*_*ji*_.

### Quantum mean first passage time

The classical notion of reaching a vertex does not have an appropriate quantum counterpart. There are some subtleties that make defining a quantum analogue a troublesome task. The main difficulty lies in the measurement problem.

Let us recall the picture with a single quantum particle wandering through a graph. Now we can imagine that we have placed a measurement apparatus at each vertex of the graph. This apparatus performs an arbitrary quantum measurement *μ*. If the quantum measurement is trivial, *i.e.* *μ*(*o*) = 𝟙_𝓗_1__, then it allows only to check whether the particle is placed in a given vertex. We can also choose a measurement that would tell us if the particle has a given property. This is done using a measurement having two values: $\Pi_{v},\Pi_{v}^{⊥}={𝟙}_{{𝓗}_{1}}-\Pi_{v}$. Hereafter, we will call Π_*v*_ the view operator.

Let us construct the following sub-TOM 𝓕 based on a given TOM 𝓔 such that:

$$\begin{array}{c} 𝓕=𝓔𝓟, \end{array}$$ (11)

where 𝓟 is diagonal sub-TOM with identity operators on the diagonal except element *P*_*jj*_, where $P_{jj}(\cdot )=\Pi_{v}^{⊥}\cdot \Pi_{v}^{⊥}$.

**Definition 17** *The quantum Mean First Passage Time (qMFPT) of (𝓔, *V*, *ρ*_0_, Π_*v*_, *i*, *j*), where 𝓔 is a TOM, *ρ*_0_ ∈ Ω(𝓗_1_) is a quantum state, Π_*v*_ is a view operator and *i*, *j* ∈ *V* is*:

$$\begin{array}{c} Q_{ij}=\sum_{t=1}^{\infty }\text{Tr}\left(\Pi_{v}\alpha_{j}^{\left(t\right)}\right)t, \end{array}$$ (12)

*α* ^(*t*)^ *is given by*:

$$\begin{array}{c} \alpha^{\left(t\right)}=𝓕\left(\alpha^{\left(t-1\right)}\right), \end{array}$$ (13)

and *α* ^(0)^ is a state vector with *ρ*_0_ at the *i*-th element and all other elements equal to zero.

**Remark 4** *When* dim𝓗_1_ = 1, *the open quantum walk reduces to a classical random walk. Therefore the introduced notion of qMFPT reduces to the classical case as well*.

**Definition 18** *The vertex-qMFPT of (𝓔, *V*, *ρ*_0_, Π_*v*_, *j*) is*:

$$\begin{array}{c} Q_{j}=\frac{\sum_{i=1,i\neq j}^{\left|V\right|}Q_{ij}}{|V|-1}, \end{array}$$ (14)

*where* *i*, *j* ∈ *V*.

**Definition 19** *The degree-qMFPT of (𝓔, *V*, *ρ*_0_, Π_*v*_, *d*), where *d* ∈ ℕ is*:

$$\begin{array}{c} Q^{\left(d\right)}=\frac{\sum_{i\in V_{d}}Q_{i}}{|V_{d}|}, \end{array}$$ (15)

*where* *V*_*d*_ ⊂ *V* *is the set of all vertices with degree* *d*.

**Definition 20** *The degree-qART of (𝓔, *V*, *ρ*_0_, Π_*v*_, *d*), where *d* ∈ ℕ is*:

$$\begin{array}{c} Q_{\text{ART}}^{\left(d\right)}=\frac{\sum_{i\in V_{d}}Q_{ii}}{|V_{d}|}, \end{array}$$ (16)

*where* *V*_*d*_ ⊂ *V* *is the set of all vertices with degree* *d*.

**Remark 5** *In an Apollonian network, vertices of a given generation have equal degrees*.

**Remark 6** *By* *T*_*j*_, *T* ^(*d*)^ *and* $T_{\text{ART}}^{\left(d\right)}$ *we denote the classical vertex-MFPT, degree-MFPT and degree-ART, respectively*.

**Remark 7** *Calculating analytically the qMFPT as shown in Definition 17 is a complicated problem. Thus, we turned to numerical simulations to obtain approximate results for a selected number of test cases. In order to numerically calculate the qMFPT, we have to limit* *t* *in Eq (12) to a finite value* *t*_s_. *We choose such a value that*:

$$\begin{array}{c} 1-\sum_{t=1}^{t_{s}}\text{Tr}\left(\Pi_{v}\alpha_{j}^{\left(t\right)}\right)<10^{-6}. \end{array}$$ (17)

## Discussion

Let us now focus our attention on the quantumness of the model introduced in the previous section. In the following sections we provide a number of examples that allow the observation of non-classical phenomena. However, it is not always the case. It is crucial to note that, with appropriate TOM design, the walk mimics a classical one.

**Remark 8** *For any open quantum walk designed with the use of unitary transformations exclusively the position probability distribution at each step of the walk is identical to the classical counterpart*.

*Moreover, the values of qMFPT and qART match the classical counterparts when the identity 𝟙 view operator is considered regardless of the initial state.*

Let us introduce a simple walk that allows tracking its evolution in detail in order to provide an example of non-classical behaviour.

### Simple example

**Fig 3:**
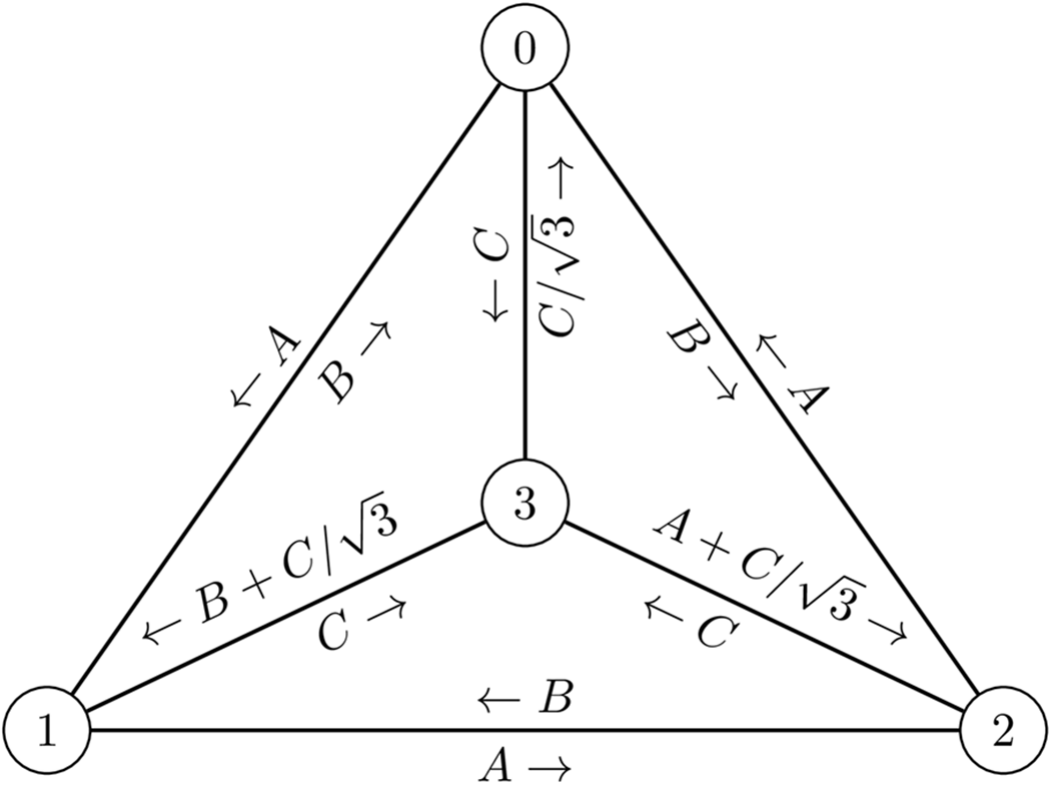
Apollonian network with 4 vertices. Operators *A*, *B* and *C* are defined in the text.

As the first example we study the four-vertex Apollonian network with a walking qutrit. This network is schematically depicted in Fig 3. We study three different view operators: *A* = ∣*x*⟩⟨*x*∣, *B* = ∣*y*⟩⟨*y*∣, *C* = ∣*z*⟩⟨*z*∣, where *A*, *B*, *C* ∈ 𝓛(ℂ^3^) and

$$\begin{array}{c} \left|x\right\rangle ={\left[1\;1\;1\right]}^{T}/\sqrt{3},\;|y\rangle ={\left[1\;\omega \;\omega^{2}\right]}^{T}/\sqrt{3},\;\left|z\right\rangle ={\left[1\;\omega^{2}\;\omega^{4}\right]}^{T}/\sqrt{3}, \end{array}$$ (18)

with *ω* = *e* ^2*π*i/3^. We choose the initial state of the walker to be the maximally mixed state localized at the central vertex

$$\begin{array}{c} \alpha^{\left(0\right)}={\left(0_{{\mathbb{C}}^{3}},0_{{\mathbb{C}}^{3}},0_{{\mathbb{C}}^{3}},\frac{1}{3}{𝟙}_{{\mathbb{C}}^{3}}\right)}^{T}. \end{array}$$ (19)

**Fig 4:**
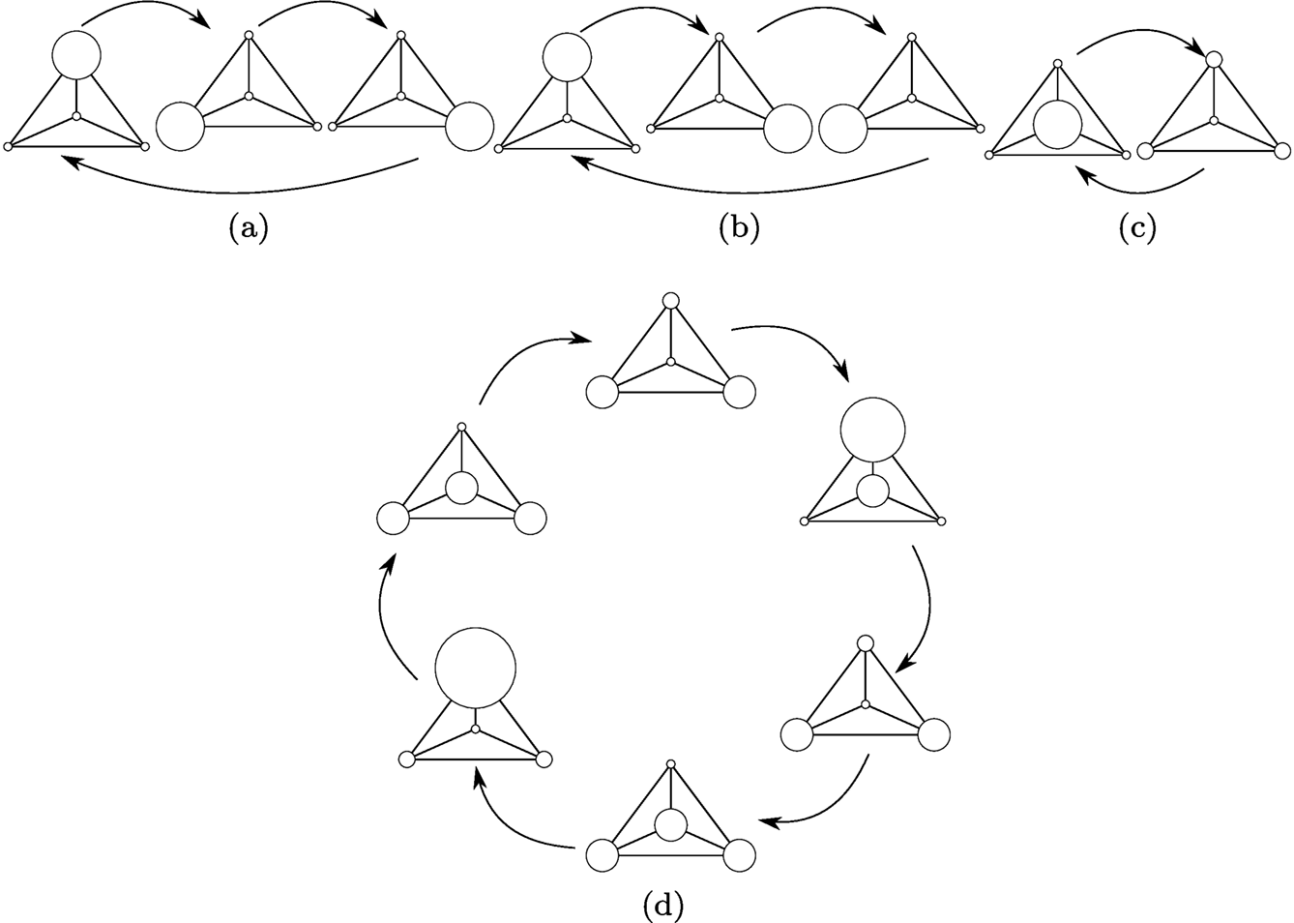
Open quantum walk on an Apollonian network with 4 vertices. Each panel of this Figure shows the behaviour of the network in subspaces associated with selected measurement operators. The size of the vertices is proportional to the probability of measuring the walker in that vertex. The picture represents the evolution after the first step of the walk. Panel (a) corresponds to Π_v_ = *A*, panel (b) to Π_v_ = *B*, panel (c) to Π_v_ = *C* and panel (d) to Π_v_ = 𝟙.

The probability distributions of measuring the particle depending on the view operators are depicted in Fig 4. Note that in the initial state the particle is located in vertex three. After the first step the behaviour becomes cyclic. Fig 4a shows the behaviour of the walker in the subspace associated with view operator *A*. Accordingly, Fig 4b shows the same result in the case of view operator *B* and Fig 4c for the operator *C*. The complete behaviour of the walker is shown in Fig 4d. In the first subspace we achieved a counter-clockwise walk on the external vertices. In the second subspace, we achieved a clockwise walk on the external vertices. Both of these walks have a period *T* = 3. In the third subspace we achieved an oscillating behaviour, between the central and external vertices so the walk has a period of *T* = 2. Thus, the entire walk is periodic with period *T* = 6. Hence we have shown that the evolution of the open quantum walk heavily depends on the view operator.

The explicit form of TOM depicted in Fig 3 reads

$$\begin{array}{c} 𝓔=[\begin{array}{cccc} 0_{𝓛({\mathbb{C}}^{3})} & B\cdot B^{†} & A\cdot A^{†} & \frac{1}{3}C\cdot C^{†}\\ A\cdot A^{†} & 0_{𝓛({\mathbb{C}}^{3})} & B\cdot B^{†} & \left(B+C/\sqrt{3}\right)\cdot {\left(B+C/\sqrt{3}\right)}^{†}\\ B\cdot B^{†} & A\cdot A^{†} & 0_{𝓛({\mathbb{C}}^{3})} & \left(A+C/\sqrt{3}\right)\cdot {\left(A+C/\sqrt{3}\right)}^{†}\\ C\cdot C^{†} & C\cdot C^{†} & C\cdot C^{†} & 0_{𝓛({\mathbb{C}}^{3})} \end{array}], \end{array}$$ (20)

where 𝓔_*ij*_(⋅) = *X*⋅*X* ^†^ denotes a rank-one quantum operation. Using 𝓔 and setting *V* = {0,1,2,3}, $\rho_{0}=\frac{1}{3}{𝟙}_{{\mathbb{C}}^{3}}$ and Π_*v*_ equal to 𝟙_ℂ^3^_, ∣0⟩⟨0∣, ∣1⟩⟨1∣, ∣2⟩⟨2∣, *A*, *B* or *C* we compute the qMFPTs and qARTs of this open quantum walk as shown in Tables 1, 2, 3, 4 and 5. Value ∞ means that the state cannot be reached from a given initial state. The diagonal entries are the qARTs. For comparison in Table 6 we show the MFPTs and ARTs in the classical homogeneous random walks on this graph. The “quantumness”—the non-classical behaviour—of the walk can be seen in the view-conditioned qMFPTs.

**Table 1:** qMFPTs from vertex *i* to vertex *j* (off-diagonal elements) and qARTs (diagonal elements) for an open quantum walk on a network shown in Fig 4 conditioned on the measurement Π_v_ = 𝟙_ℂ^3^_ and the initial state $\rho_{0}=\frac{1}{3}{𝟙}_{{\mathbb{C}}^{3}}$.

		*j*			
		0	1	2	3
*i*	0	3	3	3	1
	1	3	3	3	1
	2	3	3	3	1
	3	3	3	3	1

**Table 2:** qMFPTs from vertex *i* to vertex *j* (off-diagonal elements) and qARTs (diagonal elements) for an open quantum walk on a network shown in Fig 4 conditioned on the measurements Π_v_ = ∣0⟩⟨0∣, Π_v_ = ∣1⟩⟨1∣ or Π_v_ = ∣2⟩⟨2∣ and the initial state $\rho_{0}=\frac{1}{3}{𝟙}_{{\mathbb{C}}^{3}}$.

		*j*			
		0	1	2	3
*i*	0	5	5	5	5/4
	1	5	5	5	5/4
	2	5	5	5	5/4
	3	5	5	5	5/4

**Table 3:** qMFPTs from vertex *i* to vertex *j* (off-diagonal elements) and qARTs (diagonal elements) for an open quantum walk on a network shown in Fig 4 conditioned on the measurement Π_v_ = *A* and the initial state $\rho_{0}=\frac{1}{3}{𝟙}_{{\mathbb{C}}^{3}}$.

		*j*			
		0	1	2	3
*i*	0	2	1	2	∞
	1	2	2	1	∞
	2	1	2	2	∞
	3	2	3	1	∞

**Table 4:** qMFPTs from vertex *i* to vertex *j* (off-diagonal elements) and qARTs (diagonal elements) for an open quantum walk on a network shown in Fig 4 conditioned on the measurement Π_v_ = *B* and the initial state $\rho_{0}=\frac{1}{3}{𝟙}_{{\mathbb{C}}^{3}}$.

		*j*			
		0	1	2	3
*i*	0	2	2	1	∞
	1	1	2	2	∞
	2	2	1	2	∞
	3	2	1	3	∞

**Table 5:** qMFPTs from vertex *i* to vertex *j* (off-diagonal elements) and qARTs (diagonal elements) for an open quantum walk on a network shown in Fig 4 conditioned on the measurement Π_v_ = *C* and the initial state $\rho_{0}=\frac{1}{3}{𝟙}_{{\mathbb{C}}^{3}}$.

		*j*			
		0	1	2	3
*i*	0	5	6	6	1
	1	6	5	6	1
	2	6	6	5	1
	3	5	5	5	1

**Table 6:** MFPTs from vertex *i* to vertex *j* (off-diagonal elements) and ARTs (diagonal elements) for classical random walk on a network shown in Fig 4.

		*j*			
		0	1	2	3
*i*	0	4	3	3	3
	1	3	4	3	3
	2	3	3	4	3
	3	3	3	3	4

The walk designed in this example is based on a non-bistochastic transition operators [38]. This allows us to demonstrate the sharp non-classical behaviour of the walk that can occur in such case.

## Experiments

The discussion above gives some insight on the relation between walk behaviour and its crucial properties such as the TOM design, view operator form and the state chosen to define the starting conditions. Now we discuss a series of experiments that illustrate the possibility of obtaining non-classical behaviour in generalized open quantum walks considering these properties.

We will rate the walk quantumness in the terms of the qMFPT and qART by analysing the following cases:

- nearly classical walk where the quantum behaviour is initial state dependent,
- a walk based on a classical TOM for which the view operator determines the observed walk properties,
- an open quantum walk that exhibits strong non-classical phenomena for any view operator.

### Case 1—quantum counterpart

In this example we aim at constructing an OQW which resembles a classical random walk, differing from it only in some minor features. In order to achieve this, let us consider an open quantum walk which by construction mimics the structure of classical homogeneous random walk on an Apollonian network. By a homogeneous walk we understand a random walk for which the exit probability from every vertex in any allowed direction is equal to one over the degree of the vertex. In this example we will set the space associated with the integral degree of freedom of the walker to be a qutrit space, i. e. 𝓗_1_ = ℂ^3^. The walk is constructed in the following way:

- For every edge outgoing from vertices in the last generation of the Apollonian network, we assign TOM elements with two associated Kraus operators given by rescaled projections on two mutually orthogonal subspaces so that condition in Def. 10 holds.
- Every other TOM element is a rescaled identity operator.

Specifically, each outgoing edge of the vertices in the last generation is assigned one of the following pairs of Kraus operators:

$$\begin{array}{ccc} {𝓟}_{1} & = & (\frac{1}{\sqrt{2}}|0\rangle \langle 0|,\frac{1}{\sqrt{2}}\left|1\rangle \langle 1\right|),\\ {𝓟}_{2} & = & (\frac{1}{\sqrt{2}}|1\rangle \langle 1|,\frac{1}{\sqrt{2}}\left|2\rangle \langle 2\right|),\\ {𝓟}_{3} & = & (\frac{1}{\sqrt{2}}|2\rangle \langle 2|,\frac{1}{\sqrt{2}}\left|0\rangle \langle 0\right|). \end{array}$$ (21)

As each vertex in the last generation has degree *d* = 3, it is easily seen that this assignment fulfills Def. 10.

For the sake of clarity we will show the behavior of the walks on a 3^rd^ generation Apollonian network. In this case, we can write the assignment of pairs Eq (21) explicitly:

$$\begin{array}{cccc} E_{1,0,7} & = & \left|0\rangle \langle 0\right|/\sqrt{2},\;E_{2,0,7}=\left|1\rangle \langle 1\right|/\sqrt{2},\;E_{1,1,7}=\left|1\rangle \langle 1\right|/\sqrt{2},\;E_{2,1,7}=\left|2\rangle \langle 2\right|/\sqrt{2},\\ E_{1,4,7} & = & \left|2\rangle \langle 2\right|/\sqrt{2},\;E_{2,4,7}=\left|0\rangle \langle 0\right|/\sqrt{2},\\ E_{1,0,8} & = & \left|0\rangle \langle 0\right|/\sqrt{2},\;E_{2,0,8}=\left|1\rangle \langle 1\right|/\sqrt{2},\;E_{1,4,8}=\left|1\rangle \langle 1\right|/\sqrt{2},\;E_{2,4,8}=\left|2\rangle \langle 2\right|/\sqrt{2},\\ E_{1,3,8} & = & \left|2\rangle \langle 2\right|/\sqrt{2},\;E_{2,3,8}=\left|0\rangle \langle 0\right|/\sqrt{2}, & \\ E_{1,1,9} & = & \left|0\rangle \langle 0\right|/\sqrt{2},\;E_{2,1,9}=\left|1\rangle \langle 1\right|/\sqrt{2},\;E_{1,4,9}=\left|1\rangle \langle 1\right|/\sqrt{2},\;E_{2,4,9}=\left|2\rangle \langle 2\right|/\sqrt{2},\\ E_{1,3,9} & = & \left|2\rangle \langle 2\right|/\sqrt{2},\;E_{2,3,9}=\left|0\rangle \langle 0\right|/\sqrt{2}, & \\ E_{1,1,10} & = & \left|0\rangle \langle 0\right|/\sqrt{2},\;E_{2,1,10}=\left|1\rangle \langle 1\right|/\sqrt{2},\;E_{1,2,10}=\left|1\rangle \langle 1\right|/\sqrt{2},\;E_{2,2,10}=\left|2\rangle \langle 2\right|/\sqrt{2},\\ E_{1,5,10} & = & \left|2\rangle \langle 2\right|/\sqrt{2},\;E_{2,5,10}=\left|0\rangle \langle 0\right|/\sqrt{2},\\ E_{1,1,11} & = & \left|0\rangle \langle 0\right|/\sqrt{2},\;E_{2,1,11}=\left|1\rangle \langle 1\right|/\sqrt{2},\;E_{1,5,11}=\left|1\rangle \langle 1\right|/\sqrt{2},\;E_{2,5,11}=\left|2\rangle \langle 2\right|/\sqrt{2},\\ E_{1,3,11} & = & \left|2\rangle \langle 2\right|/\sqrt{2},\;E_{2,3,11}=\left|0\rangle \langle 0\right|/\sqrt{2},\\ E_{1,2,12} & = & \left|0\rangle \langle 0\right|/\sqrt{2},\;E_{2,2,12}=\left|1\rangle \langle 1\right|/\sqrt{2},\;E_{1,5,12}=\left|1\rangle \langle 1\right|/\sqrt{2},\;E_{2,5,12}=\left|2\rangle \langle 2\right|/\sqrt{2},\\ E_{1,3,12} & = & \left|2\rangle \langle 2\right|/\sqrt{2},\;E_{2,3,12}=\left|0\rangle \langle 0\right|/\sqrt{2},\\ E_{1,0,13} & = & \left|0\rangle \langle 0\right|/\sqrt{2},\;E_{2,0,13}=\left|1\rangle \langle 1\right|/\sqrt{2},\;E_{1,2,13}=\left|1\rangle \langle 1\right|/\sqrt{2},\;E_{2,2,13}=\left|2\rangle \langle 2\right|/\sqrt{2},\\ E_{1,6,13} & = & \left|2\rangle \langle 2\right|/\sqrt{2},\;E_{2,6,13}=\left|0\rangle \langle 0\right|/\sqrt{2},\\ E_{1,2,14} & = & \left|0\rangle \langle 0\right|/\sqrt{2},\;E_{2,2,14}=\left|1\rangle \langle 1\right|/\sqrt{2},\;E_{1,6,14}=\left|1\rangle \langle 1\right|/\sqrt{2},\;E_{2,6,14}=\left|2\rangle \langle 2\right|/\sqrt{2},\\ E_{1,3,14} & = & \left|2\rangle \langle 2\right|/\sqrt{2},\;E_{2,3,14}=\left|0\rangle \langle 0\right|/\sqrt{2},\\ E_{1,0,15} & = & \left|0\rangle \langle 0\right|/\sqrt{2},\;E_{2,0,15}=\left|1\rangle \langle 1\right|/\sqrt{2},\;E_{1,6,15}=\left|1\rangle \langle 1\right|/\sqrt{2},\;E_{2,6,15}=\left|2\rangle \langle 2\right|/\sqrt{2},\\ E_{1,3,15} & = & \left|2\rangle \langle 2\right|/\sqrt{2},\;E_{2,3,15}=\left|0\rangle \langle 0\right|/\sqrt{2}. \end{array}$$ (22)

The numbering of vertices follows the convention shown in Fig 1.

In this case we use the following initial state for calculating degree-qMFPT and degree-qART: *ρ*_0_ = ∣*x*⟩⟨*x*∣, where $|x\rangle ={\left[1\;1\;1\right]}^{\text{T}}/\sqrt{3}$. For such an initial state we obtain the results differing from classical ones even when the view operator is equal to the identity Π_*v*_ = 𝟙_𝓗_1__. The resulting degree-qMFPTs and degree-qARTs are shown in Tables 7 and 8 respectively. The most significant difference from the classical set-up is that the degree-qARTs do not scale as $\frac{1}{d}$, where *d* is the degree of the vertex. It should be noted that by the construction of the model, when the initial state is a classical mixture $\rho_{0}=\frac{1}{3}{𝟙}_{{\mathbb{C}}^{3}}$, we can recover the classical behaviour.

**Table 7:** Degree-(q)MFPTs for the classical and quantum for a walk on the Apollonian network of the third generation. The TOM assignment is described in the text. Here, we put Π_*v*_ = 𝟙_ℂ^3^_, *ρ*_0_ = ∣*x*⟩⟨*x*∣. We obtain the behaviour different from the classical case.

	*d*			
	3	6	9	12
Classical	30.35	15.87	9.47	6.33
Quantum	30.77	16.53	10.12	7.27

**Table 8:** Degree-(q)ARTs for the classical and quantum for a walk on the Apollonian network of the third generation. The TOM assignment is described in the text. Here, we put Π_*v*_ = 𝟙_ℂ^3^_, *ρ*_0_ = ∣*x*⟩⟨*x*∣. We obtain the behaviour different from the classical case. Notice that the degree-qART does not scale as $\frac{1}{d}$.

	*d*			
	3	6	9	12
Classical	28.00	14.00	9.33	7.00
Quantum	27.25	13.63	9.00	6.97

In the case of a walk defined mostly with the use of the identity operators the vast majority of the transition operations is bi-stochastic. The disturbance is introduced in the last generation nodes. We have shown that with a slight change of the walk structure and with an appropriate initial state we observe a significant alteration of the walk and non-classical behaviour, even without considering the view operator.

### Case 2—measurement manipulation

In this case we aim to analyse the behaviour of a walk based on bi-stochastic operations exclusively. In particular we investigate the behaviour in terms of qMFTP and qART values when a variety of view operators is applied. We consider an open quantum walk on the fifth generation Apollonian network, constructed as follows.

Let *d*_*i*_ be the degree of vertex *i*, the internal state space to be two-dimensional 𝓗_1_ = ℂ^2^, *G*_1_, *G*_2_ with *G*_1_ > *G*_2_ denote two different generations of the Apollonian networks, *V*_*G*_1__ and *V*_*G*_2__ be the sets of vertices in generations *G*_1_ and *G*_2_, respectively. By *i* and *j* we denote vertices in generations *G*_1_ and *G*_2_, respectively, *i.e.* *i* ∈ *V*_*G*_1__, *j* ∈ *V*_*G*_2__. Then:

- For transitions from generation *G*_1_ to generation *G*_2_, we choose TOM elements equal to ${𝓔}_{ji}(\rho )=\frac{1}{d}\sigma_{x}\rho \sigma_{x}$.
- For transitions from generation *G*_2_ to generation *G*_1_ we choose TOM elements equal to ${𝓔}_{ij}(\rho )=\frac{1}{d}\sigma_{z}\rho \sigma_{z}$.
- In the case of the zeroth generations there exist intra-generation transitions. Let us denote by *k*, *l* ∈ *V*_*G*_0__ the vertices in this generation. For these transitions we assign a rescaled identity operator ${𝓔}_{kl}(\rho )=\frac{1}{d}\rho$.

Here, *σ*_*x*_ and *σ*_*z*_ are the Pauli matrices given by:

$$\begin{array}{c} \sigma_{x}=(\begin{array}{cc} 0 & 1\\ 1 & 0 \end{array}),\;\sigma_{z}=(\begin{array}{cc} 1 & 0\\ 0 & -1 \end{array}). \end{array}$$ (23)

We use the following view operators Π_*v*_: 𝟙_ℂ^2^_, ∣0⟩⟨0∣, ∣+⟩⟨+∣ and ∣*j*⟩⟨*j*∣, with:

$$\begin{array}{ccc} \left|+\right\rangle & = & \frac{1}{\sqrt{2}}(|0\rangle +|1\rangle ),\\ \left|j\right\rangle & = & \frac{1}{\sqrt{2}}(|0\rangle +i|1\rangle ). \end{array}$$ (24)

In this case we choose the initial state to be $\rho_{0}=\frac{1}{2}{𝟙}_{{\mathbb{C}}^{2}}$.

**Fig 5:**
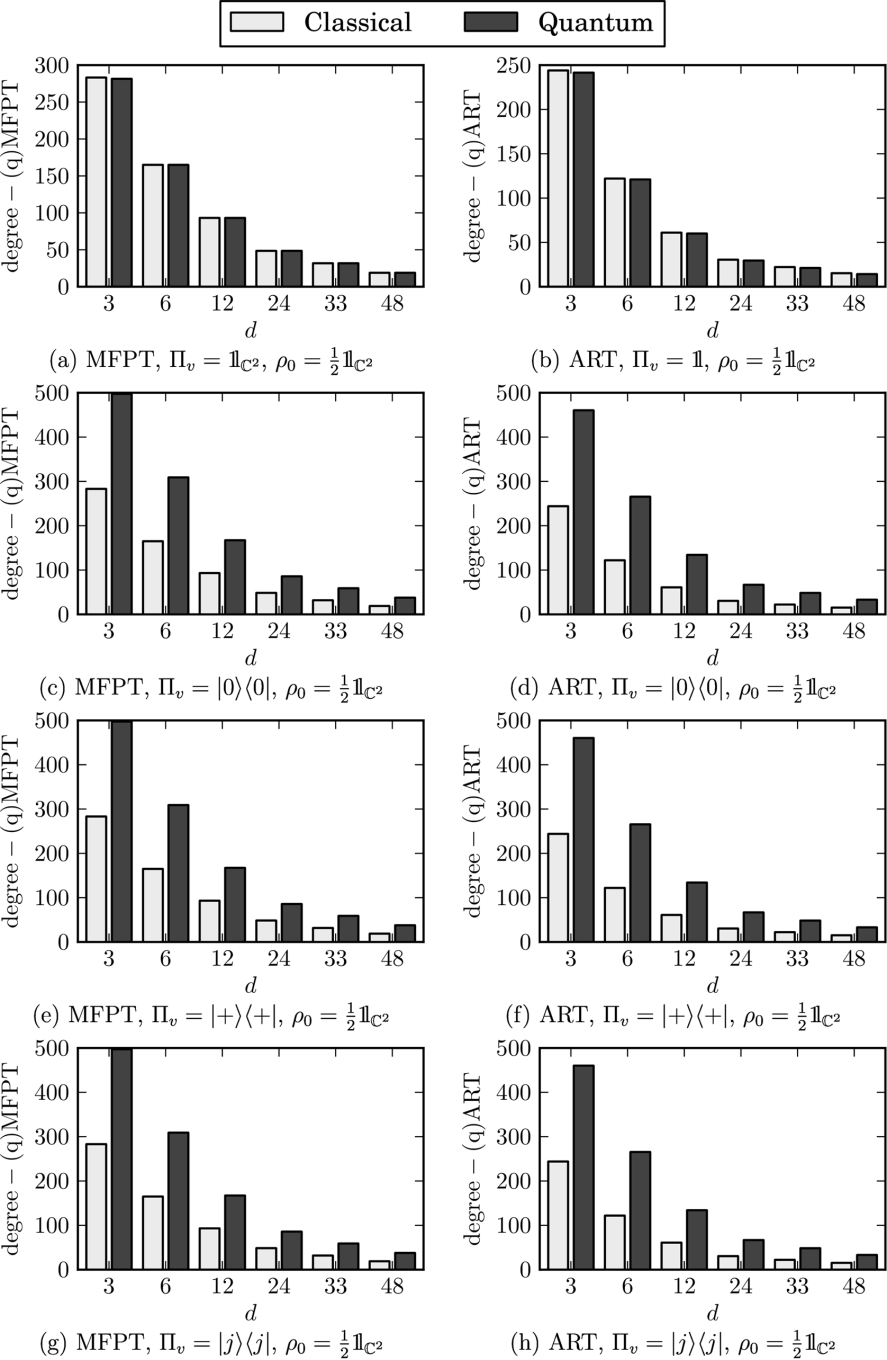
Degree-(q)MFPTs and degree-(q)ARTs conditioned on the view operator. The labels denote the conditioning measurement operators.

The results for this case are shown in Fig 5. As all of the transition operators are bi-stochastic, the walk exhibits exactly classical behaviour when the view operator equals to 𝟙_ℂ^2^_. Although the view operators increase the value of degree-qMFPT, the values differ only by a constant factor. Hence the overall trend remains unchanged. As in the previous case, the main difference between the classical and quantum set-ups lies in the degree-qARTs. Again, they do not scale as $\frac{1}{d}$. Thus, the view operator is the key ingredient that allows the observation of non-classical behaviour in the case of a walk with bi-stochastic transition operations.

### Case 3—quantum effect

In this case we again consider non-bistochastic walk. The walk does not mimic a classical one and thus we are able to obtain striking differences in terms of MFPT/ART behaviour. In this case the transition operator assignment is also based on the generation of a vertex. More precisely, we divide vertices in the graph into classes. Each class is identified by the set of generations of the neighbouring vertices. As a result, each class corresponds to the vertices with identical configuration of generations of neighbouring vertices.

**Fig 6:**
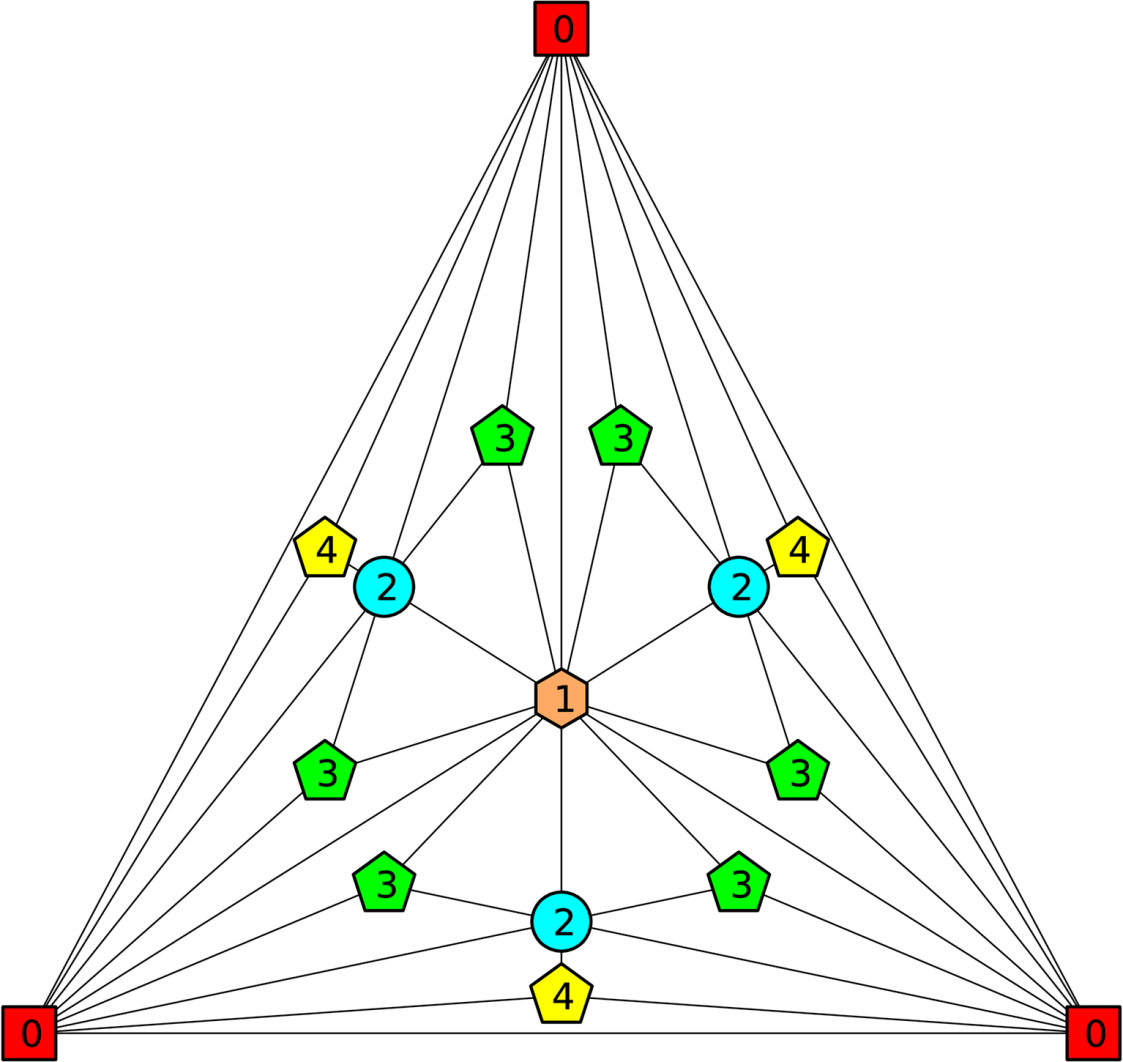
The Apollonian network with 16 vertices (3^rd^ generation) divided into 5 classes. The classes are chosen based on the vertex generation and the generations of its neighbours. In this case, the green pentagons and yellow pentagons are of the same generation, but belong to different classes. The numbers denote the classes used in Eq (27).

Here we consider the 3^rd^ generation of Apollonian network consisting of 16 vertices divided into 5 classes as shown in Fig 6. This approach allows us the simplified assignment of operators, as the number of classes is significantly lower than the number of vertices and provides strong symmetry of the network dynamics.

In order to design system dynamics we introduce two decompositions of the space 𝓗_1_ into mutually orthogonal subspaces. For each subspace we choose an operator that acts on this subspace exclusively. In this example, we set 𝓗_1_ = ℂ^4^. We study two decompositions (*x* and *z*) of 𝓗_1_ with the following operators:

$$\begin{array}{c} B_{x}=\left({𝟙}_{{\mathbb{C}}^{4}}-\sigma_{x}⊗\sigma_{x}\right)/2,\;C_{x}=\left({𝟙}_{{\mathbb{C}}^{4}}+\sigma_{x}⊗\sigma_{x}\right)/2, \end{array}$$ (25)

and

$$\begin{array}{c} B_{z}=\left({𝟙}_{{\mathbb{C}}^{4}}-\sigma_{z}⊗\sigma_{z}\right)/2,\;C_{z}=\left({𝟙}_{{\mathbb{C}}^{4}}+\sigma_{z}⊗\sigma_{z}\right)/2. \end{array}$$ (26)

For every possible pair of classes (*c*_1_, *c*_2_) we choose a set (with one or two elements in this case) of Kraus operators $\left\{A_{1}^{\left(c_{1},c_{2}\right)},\ldots ,A_{n_{c_{1},c_{2}}}^{\left(c_{1},c_{2}\right)}\right\}$ from {*B*_*x*_, *C*_*x*_, *B*_*z*_, *C*_*z*_}. In order to satisfy Def. 10, we design transition operators assignment with two normalization rules. When the designed assignment causes that, for some vertex in the network, there are (*k*) outgoing edges assigned with the same operator all these operators are multiplied by the factor $1/\sqrt{k}$. For example each vertex of the class 1 has 6 outgoing edges corresponding to the *C*_*z*_ operator: 3 neighbours of class 0 and 3 neighbours of class 2. Thus we introduce $1/\sqrt{6}$ normalization factor for the $A_{1}^{\left(1,0\right)}$ and $A_{1}^{\left(1,2\right)}$ operators as shown in Eq (27). Secondly, when the operators assigned as outgoing from some class *c* correspond to two independent subspace decompositions all operators are additionally multiplied by the factor $1/\sqrt{2}$. In this case classes 0 and 2 utilize both *x* and *z* decomposition operators and thus the factor is present in $A_{k}^{\left(0,j\right)}$ and $A_{k}^{\left(2,j\right)}$ operators.

This gives us the following operator assignment, where *A* ^(*i*, *j*)^ is the operator assigned to every transition from class *i* to *j*:

$$\begin{array}{ccc} A_{1}^{\left(0,1\right)} & = & B_{z}/2,\;A_{1}^{\left(1,0\right)}=C_{z}/\sqrt{6},\;A_{1}^{\left(1,2\right)}=C_{z}/\sqrt{6},\;A_{1}^{\left(2,1\right)}=B_{x}/2,\\ A_{1}^{\left(0,2\right)} & = & C_{x}/2,\;A_{1}^{\left(2,0\right)}=C_{z}/2,\;A_{1}^{\left(1,3\right)}=B_{z}/\sqrt{6},\;A_{1}^{\left(3,1\right)}=B_{x},\\ A_{1}^{\left(2,3\right)} & = & B_{x}/\sqrt{8},\;A_{1}^{\left(3,2\right)}=C_{x}/\sqrt{2},\;A_{1}^{\left(0,3\right)}=B_{z}/\sqrt{8},\;A_{1}^{\left(3,0\right)}=C_{x}/\sqrt{2},\\ A_{1}^{\left(2,4\right)} & = & B_{z}/\sqrt{2},\;A_{2}^{\left(2,4\right)}=C_{x}/\sqrt{2},\;A_{1}^{\left(4,2\right)}=C_{x},\;\\ A_{1}^{\left(0,4\right)} & = & C_{z}/2,\;A_{2}^{\left(0,4\right)}=B_{x}/\sqrt{8},\;A_{1}^{\left(4,0\right)}=B_{x}/\sqrt{2},\;\\ A_{1}^{\left(0,0\right)} & = & B_{x}/\sqrt{8}.\; \end{array}$$ (27)

The class numbers correspond to those shown in Fig 6. The initial state is $\rho_{0}=\frac{1}{4}{𝟙}_{{\mathbb{C}}^{4}}$.

**Fig 7:**
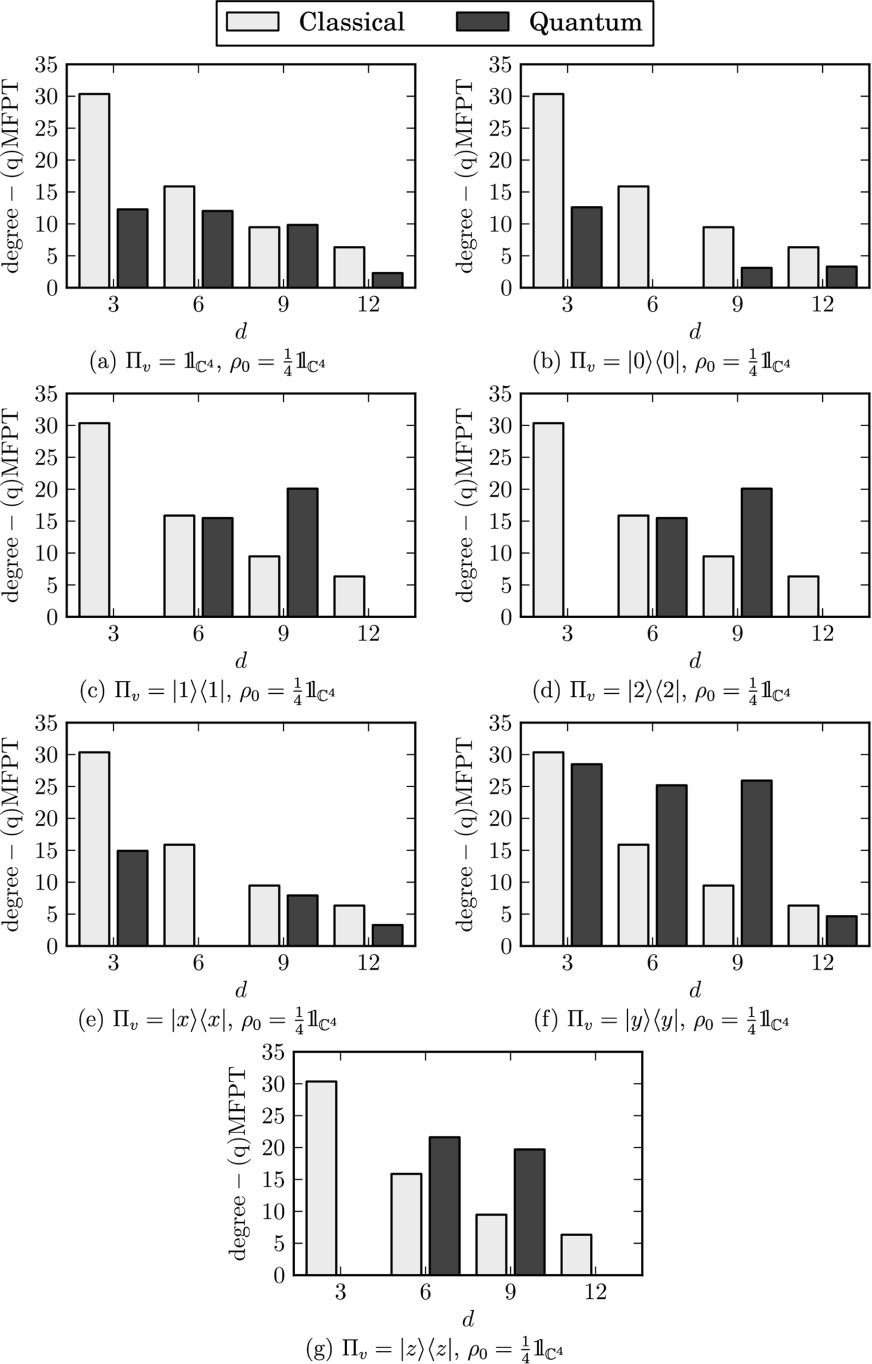
Degree-(q)MFPTs conditioned on the view operator. A missing bar indicates that the appropriate vertices are unreachable under the given view operator. Panel (a) Π_*v*_ = 𝟙, panel (b) Π_*v*_ = ∣0⟩⟨0∣, panel (c) Π_*v*_ = ∣1⟩⟨1∣, panel (d) Π_*v*_ = ∣2⟩⟨2∣, panel (e) Π_*v*_ = ∣*x*⟩⟨*x*∣, panel (f) Π_*v*_ = ∣*y*⟩⟨*y*∣, panel (g) Π_*v*_ = ∣*z*⟩⟨*z*∣.

**Fig 8:**
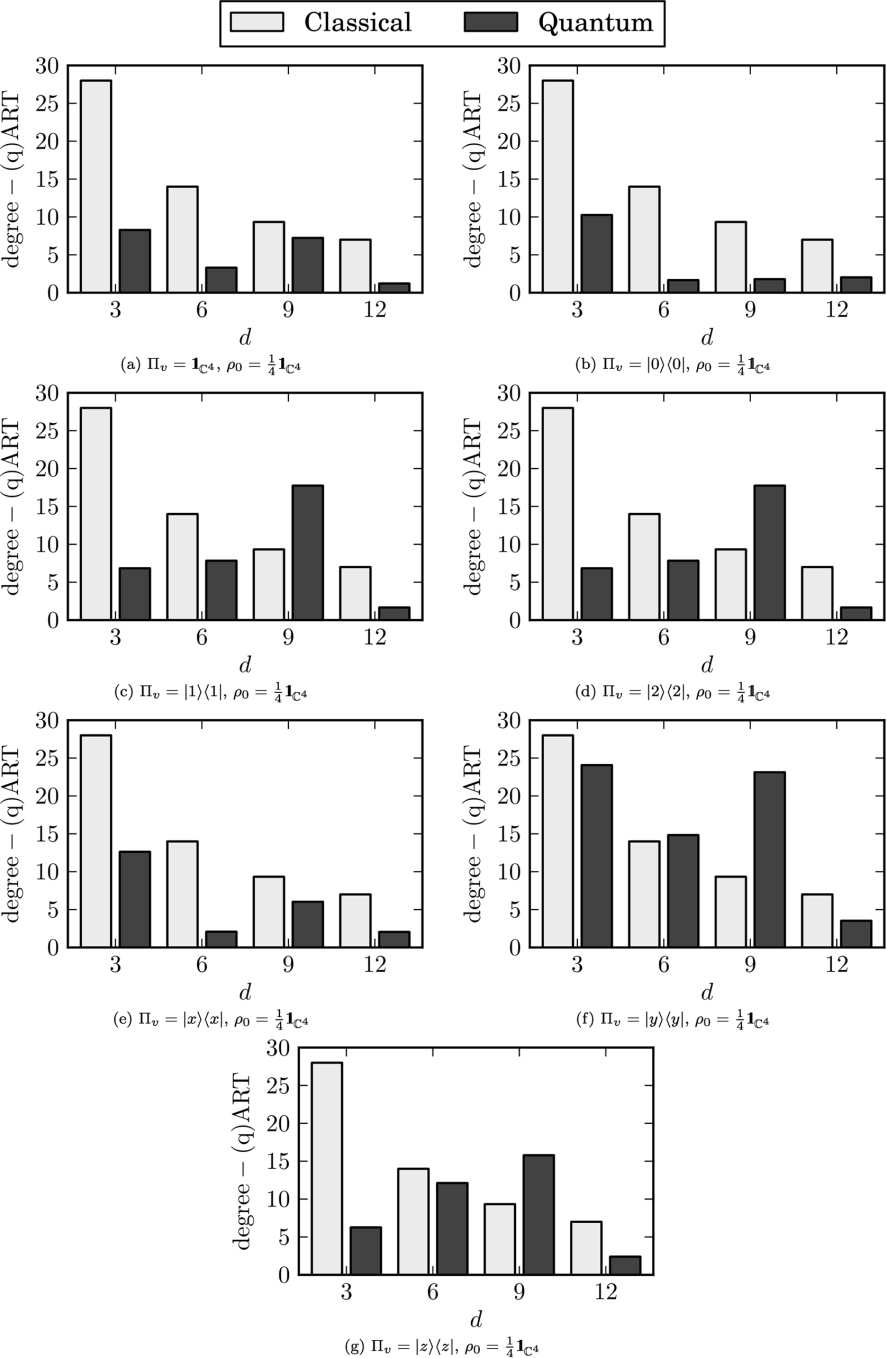
Degree-(q)ARTs conditioned on the view operator. Panel (a) Π_*v*_ = 𝟙, panel(b) Π_*v*_ = ∣0⟩⟨0∣, panel(c) Π_*v*_ = ∣1⟩⟨1∣, panel(d) Π_*v*_ = ∣2⟩⟨2∣, panel(e) Π_*v*_ = ∣*x*⟩⟨*x*∣, panel(f) Π_*v*_ = ∣*y*⟩⟨*y*∣, panel(g) Π_*v*_ = ∣*z*⟩⟨*z*∣.

The numerical results are shown in Figs 7 and 8. This time we obtain qMFPTs conditioned on the view operator which are significantly different from the classical ones. Furthermore, the ARTs are no longer monotonic functions of the vertex degree *d* and the non-classicality is present regardless of the view operator. Moreover, some positions become unreachable when the view operator is applied.

## Conclusions

The main contribution of this work is the introduction of a generalized model of open quantum walks, that is derived from the idea of Quantum Markov Chains. We apply this model to study the evolution of quantum walks on Apollonian networks that provides some insight on the role of the network properties on the resulting quantum dynamics. We have also provided definitions of mean first passage time and average return time for generalized open quantum walks. We have calculated these quantities for several examples and compared them with the classical case.

We have shown illustrative set-ups of exciton transport in Apollonian networks which can lead to very non-trivial behaviour compared to ordinary quantum walks. In some cases we are able to recover the classical behaviour, although in general the model allows for much richer walker behaviour. Hence, the open quantum walk model can be used to explain non-trivial behaviour not only in linear, but also in more complex topologies of the underlying graphs. Furthermore, we have studied mean first passage times and average return times in this set-up. These results differ significantly from a classical walk on these networks. The results allow us theeasy creation of walks that visit certain vertices after a given time or omit a selected subset of vertices.
